# Dermatomyositis-like Eruption Induced by Hydroxyurea—Case Report and Literature Review

**DOI:** 10.3390/jcm14072192

**Published:** 2025-03-23

**Authors:** Loredana Elena Stoica, Mihaela Roxana Mitroi, Oana Maria Ică, Alina Maria Vîlcea, Lavinia Petruța Fronie-Andrei, Cristina Ioana Vîlcea, Raluca Niculina Ciurea, Andreea Mihai, George G. Mitroi

**Affiliations:** 1 Department of Dermatology, Faculty of Medicine, University of Medicine and Pharmacy of Craiova, 200349 Craiova, Romaniamitroi.george@yahoo.com (G.G.M.); 2 Department of Otorhinolaryngology, Faculty of Medicine, University of Medicine and Pharmacy of Craiova, 200349 Craiova, Romania; 3 Department of Pathology, University of Medicine and Pharmacy of Craiova, 200349 Craiova, Romania; 4 Department of Pulmonology, University of Medicine and Pharmacy of Craiova, 200349 Craiova, Romania

**Keywords:** hydroxyurea, dermatomyositis, adverse effect

## Abstract

**Background:** Hydroxyurea (HU) is a widely used chemotherapeutic agent for myeloproliferative disorders, yet its long-term use can rarely trigger a dermatomyositis-like (DM-like) eruption characterized solely by cutaneous manifestations without muscle involvement or serologic markers. This study presents a case of HU-induced DM-like eruption and reviews the literature regarding this rare occurrence. **Methods:** A 77-year-old woman with polycythemia vera on long-term HU therapy developed a progressively worsening, erythematous, scaly, and crusted eruption on the face, neck, and anterior thorax. Comprehensive clinical evaluations, laboratory tests (including normal muscle enzymes and negative autoimmune panels), and skin biopsies were performed. In parallel, a systematic literature review was conducted using databases such as PubMed, Scopus, and Google Scholar, incorporating case reports and series published prior to January 2025 that provided detailed individual clinical data. **Results:** The patient exhibited hallmark DM-like cutaneous features—interface dermatitis with basal vacuolar degeneration and prominent dermal mucin deposition—without evidence of muscle weakness or positive myositis-specific antibodies. The literature review of 23 cases revealed a median latency of 5 years from HU initiation to skin eruption, with the dorsal hands most frequently affected. HU discontinuation, often combined with systemic and topical corticosteroids (and, in some cases, steroid-sparing agents), resulted in lesion resolution in over 90% of cases, with a median healing time of approximately 3 months. **Conclusions:** HU-induced DM-like eruption, though infrequent, is a distinct clinical entity requiring prompt recognition and management. The main treatment is the discontinuation of HU, which, when supplemented by appropriate corticosteroid therapy, leads to significant clinical improvement. Ongoing dermatologic surveillance is recommended for patients on long-term HU therapy due to the potential risk of premalignant skin changes.

## 1. Introduction

Hydroxyurea (HU) is a chemotherapeutic agent primarily used in the treatment of myeloproliferative disorders (MPDs). Although generally well tolerated, HU is associated with a wide range of cutaneous side effects, varying from mild to severe. Common cutaneous side effects include hyperpigmentation, xerosis, and skin atrophy, while severe complications such as leg ulcers and non-melanoma skin cancers occur in a significant number of patients and often resolve with HU discontinuation and appropriate management. Among these, DM-like eruptions are among the rarest, with only a few cases reported in the literature [1,2,3].

HU exerts its effects by inhibiting ribonucleotide reductase, specifically targeting the M2 subunit, which disrupts the synthesis of deoxyribonucleotides and prevents DNA replication. This leads to cell cycle arrest at the G_1_/S phase, impairing DNA repair mechanisms and increasing cellular susceptibility to radiation-induced damage [4]. The drug is typically administered at doses between 1 and 2 g per day, depending on the underlying condition being treated [5].

The exact mechanism behind HU-induced cutaneous toxicity is not fully understood but is believed to result from its cytotoxic impact on rapidly proliferating cells [6]. Reports on the prevalence of dermatologic side effects vary considerably, with some studies indicating an occurrence between 5 and 35%, while others suggest rates as high as 65–96% [5,6,7,8,9,10,11].

While long-term HU therapy is more commonly associated with skin-related complications, some cases have been documented within just two weeks of treatment initiation. The resolution of these side effects depends on the specific reaction and whether HU is discontinued. Serious complications such as ulcerations or skin malignancies tend to be observed in patients with MPDs receiving higher doses over prolonged periods [5].

In this paper, the term “dermatomyositis-like” refers exclusively to the cutaneous manifestations of DM, characterized by histopathological findings consistent with DM, but without muscle involvement or laboratory abnormalities indicative of classic, full-spectrum DM.

## 2. Case Report

A 77-year-old retired female with a seven-year history of polycythemia vera, managed with hydroxyurea since the time of diagnosis, presented with a progressively worsening cutaneous eruption. Her medical history included grade 3 arterial hypertension and chronic ischemic cardiopathy, for which she had been taking trimetazidine, candesartan, metoprolol, acetylsalicylic acid, and indapamide for approximately 15 years. The skin eruption began approximately one year prior, initially as erythematous plaques on the face. Despite the application of various unspecified topical therapies, the lesions progressively extended to the neck and anterior chest.

### 2.1. Clinical Examination

**Figure 1:**
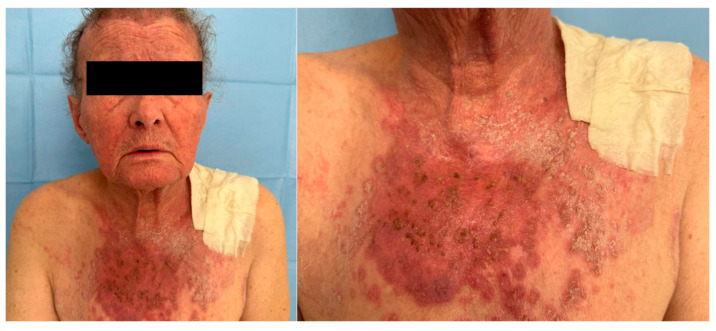
Erythematous, scaly, and crusted eruption formed by plaques and confluent infiltrated placards with relatively well-defined margins.

On physical examination, the patient exhibited an erythematous, scaly, and crusted eruption formed by plaques and confluent infiltrated placards with relatively well-defined margins. The lesions were mildly pruritic and localized circumferentially on the neck, as well as on the face and anterior thorax (Figure 1). There was no mucosal involvement, muscle weakness, or other systemic symptoms.

Upon admission, the patient’s general condition was moderate. She had a body weight of 45 kg and a height of 155 cm. She had no personal or family history of autoimmune disease or connective tissue disorders, and no significant internal medical conditions were noted. Respiratory, gastrointestinal, and renal assessments showed no abnormalities. Her blood pressure was 130/80 mmHg, and her heart rate was 78 bpm, which was regular. Cardiac auscultation revealed normal heart sounds with no murmurs or abnormal findings. The respiratory examination was unremarkable, with symmetrical lung expansion and clear breath sounds. Abdominal palpation showed no hepatosplenomegaly or tenderness. Neurological examination was within normal limits, with no signs of muscle weakness or sensory deficits.

The initial presumptive diagnosis was a drug-induced cutaneous eruption. However, given the atypical presentation of the skin lesions and their progressive evolution, a literature search was conducted to identify rare dermatological manifestations associated with the patient’s current pharmacological regimen. Based on the findings, an initial presumptive diagnosis of a DM-like eruption secondary to HU therapy was established.

### 2.2. Routine Blood Tests Were Within Normal Limits

- CBC: No leukocytosis, hemoglobin, and platelet counts within normal range, indicating successful treatment with HU.
- Liver and Kidney Function Tests: No abnormalities.
- Inflammatory Markers: C-Reactive Protein (CRP) and Erythrocyte Sedimentation Rate (ESR) (within normal range).
- Creatine Kinase (CK) and Aldolase: Normal, ruling out significant muscle involvement.
- Autoimmune Panel: Antinuclear Antibody (ANA)-negative; myositis-specific antibodies not detected.

### 2.3. Histopathology and Additional Workup

For our patient, electromyography (EMG) was performed on the deltoid, biceps brachii, quadriceps, and iliopsoas muscles—key muscle groups typically affected in DM. The results showed no evidence of myopathic changes, supporting the absence of neuromuscular involvement.

**Figure 2:**
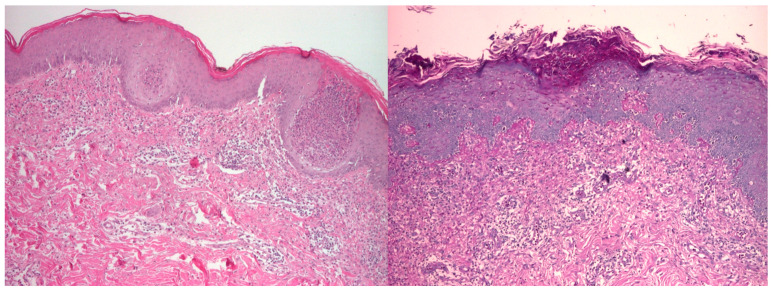
Skin biopsy at 40× magnification. (**Left**) H&E staining showing interface dermatitis (epidermal atrophy, basal vacuolar degeneration, and perivascular lymphocytic infiltrate). (**Right**) AB-PAS staining showing prominent dermal mucin deposition.

A skin biopsy was performed on the anterior thoracic region. Direct immunofluorescence was negative for immune deposits. Histopathological description: The Hematoxylin and Eosin (H&E) stained section (Figure 2 left, 40× magnification) shows characteristic features of DM-like interface dermatitis. The epidermis is atrophic, with basal vacuolar degeneration and mild hyperkeratosis, and the dermal-epidermal junction appears disrupted, with a lymphocytic inflammatory infiltrate scattered around superficial dermal blood vessels. Dermal mucin deposition is evident as pale-staining areas interspersed between collagen fibers. The papillary dermis also exhibits mild edema, further supporting a diagnosis consistent with a DM-like eruption (Figure 2, left).

The Alcian Blue–Periodic Acid Schiff (AB-PAS) stained section (Figure 2, right, 40× magnification) confirms the presence of prominent dermal mucin deposition, highlighted in blue. Inflammatory cells are scattered around perivascular and periadnexal regions, composed predominantly of mononuclear cells, without evidence of vasculitic changes. The absence of leukocytoclastic vasculitis or fibrinoid necrosis of vessels rules out alternative vasculitic processes (Figure 2, right).

These histopathological findings are consistent with a drug-induced DM-like eruption.

A dermatologic life quality index (DLQI) score was calculated at 16, reflecting a moderate impairment of quality of life.

Given the known association between DM-like eruptions and paraneoplastic syndromes, a comprehensive malignancy screening was performed, including thoracoabdominal, head, and pelvic CT imaging. No evidence of underlying malignancy was found, supporting the conclusion that the patient’s cutaneous reaction was drug-induced rather than a paraneoplastic phenomenon.

### 2.4. Diagnosis and Management

Based on the clinical presentation, histopathological findings, and exclusion of other etiologies, the patient was diagnosed with an HU-induced DM-like eruption.

Other differential diagnoses were carefully considered, but the clinical presentation and investigative findings supported an HU-induced DM-like eruption. Autoimmune DM was ruled out due to the absence of muscle symptoms, a negative autoimmune panel, and normal muscle enzyme levels (CK and aldolase), while the biopsy findings were more suggestive of a drug-induced reaction rather than an idiopathic autoimmune process. Paraneoplastic DM in the context of polycythemia vera was considered; however, the DM-like eruption was more likely drug-induced, as it showed no correlation with the disease. A reaction to other medications was deemed unlikely, as the patient had been on her cardiovascular medications for 15 years without incident, while HU was the only newly implicated drug. Similarly, HU-induced leukocytoclastic vasculitis was excluded due to the absence of palpable purpura and the lack of vascular involvement upon histopathology. Other common dermatologic conditions such as psoriasis, eczema, or a nonspecific drug eruption were also considered; however, the chronicity of the eruption, its specific distribution, and the histopathological findings were more consistent with a DM-like reaction to HU.

HU was discontinued, and the patient was started on systemic corticosteroids (prednisone 0.5 mg/kg/day) and topical corticosteroids (clobetasol propionate 0.05% bid). Adjunctive emollient therapy was recommended to improve skin barrier function. Azathioprine (2 mg/kg/day) was introduced two weeks after initiating prednisone as a steroid-sparing agent, allowing for the gradual tapering of corticosteroid therapy. Prednisone was tapered by 5 mg per week starting in week 2 until a dose of 5 mg/day was reached. Given the discontinuation of HU, the patient was referred to hematology for evaluation and selection of an alternative therapy.

Azathioprine was selected as a steroid-sparing agent due to its well-documented efficacy in autoimmune and drug-induced inflammatory dermatoses, allowing for gradual corticosteroid tapering while minimizing the risks associated with long-term steroid use. Given the patient’s advanced age and comorbidities, alternative agents such as methotrexate or mycophenolate mofetil were considered but deemed less favorable due to potential hepatotoxicity and immunosuppressive burden.

At the six-week follow-up, the lesions showed marked improvement, with a reduction in erythema and scaling. Prednisone had been tapered to 5 mg/day and was discontinued at this visit, while azathioprine (2 mg/kg/day) was continued as maintenance therapy along with topical corticosteroids and emollients. By three months, the eruption had almost completely resolved, but mild skin atrophy persisted in the affected area, and azathioprine was tapered and stopped over the following three months, with no recurrence of lesions. The patient experienced no treatment-related side effects.

## 3. Discussion

### 3.1. Overview of Hydroxyurea-Induced Dermatomyositis-like Eruption

DM is defined by its characteristic skin manifestations, including Gottron’s papules (erythematous plaques over the extensor surfaces of the hands, knees, elbows, and ankles), heliotrope rash (violaceous discoloration with periorbital edema), shawl sign (erythematous eruption over the sun-exposed upper back), V sign (erythematous rash on the upper chest), Holster sign (erythematous patches on the lateral hips and thighs), and mechanic’s hands (hyperkeratosis, fissuring of the palms and fingertips with irregular, thickened cuticles). The facial heliotrope rash appears as a symmetrical, pruritic, violaceous erythema with edema, primarily affecting the upper eyelids.

Laboratory findings often include elevated skeletal muscle enzymes such as CK, aldolase, aspartate aminotransferase (AST), alanine transaminase (ALT), and lactate dehydrogenase (LDH) [12,13]. More recently, myositis-specific autoantibodies (MSAs) and myositis-associated antibodies (MAAs) have been recognized as valuable diagnostic markers, helping to correlate specific serological profiles with clinical phenotypes of the disease [13]. However, in our patient, none of the typical laboratory abnormalities were present, with normal muscle enzyme levels (CK, aldolase, AST, ALT, and LDH) and no detectable autoantibodies, further supporting the absence of systemic muscle involvement. For a direct comparison of classic autoimmune DM and HU-induced DM-like eruption, see Table 1.

Nevertheless, up to 20% of patients with DM exhibit no overt muscle involvement or only subclinical muscle alterations; however, underlying muscle involvement can often be detected through magnetic resonance imaging (MRI), EMG, or muscle biopsy [14,15]. For our patient, EMG was performed and yielded normal results, indicating no detectable muscle involvement.

Regarding paraneoplastic dermatomyositis (PDM), this subtype is characterized by its association with an underlying malignancy. Patients diagnosed with DM may develop an occult malignancy, and various studies report widely differing prevalence rates, with neoplasia detected in up to 40% of cases [16]. However, it was unlikely that, in our case, the patient’s DM-like eruption represented a paraneoplastic phenomenon associated with polycythemia vera, rather than a drug-induced reaction, as the skin lesions did not correlate with the progression of the hematologic malignancy and resolved within 12 weeks after discontinuation of HU.

### 3.2. Single- and Two-Patient Case Reports

A literature search was performed using databases such as PubMed, Scopus, and Google Scholar, using key terms including “dermatomyositis”, “hydroxyurea”, “drug-induced dermatomyositis”, “hydroxyurea adverse effects”, “cutaneous toxicity”, and “hydroxyurea skin reactions”. The inclusion criteria for this study encompassed case reports, case series, and reviews describing HU-induced DM-like eruptions published before 31 January 2025. Only cases with sufficient clinical details, including patient demographics, underlying conditions, duration of HU therapy, clinical presentation, histopathological findings, and management outcomes, were included. Studies focusing on classic autoimmune DM, drug reactions unrelated to HU, or cases lacking adequate documentation were excluded. Additionally, broader reviews or meta-analyses that mentioned HU-induced DM but did not provide specific case details were not considered.

Inclusion Criteria:
  - Case reports, case series, and reviews describing HU-induced DM-like eruptions.
  - Studies published before 31 January 2025.
  - Articles providing detailed clinical data (patient demographics, underlying condition, duration of HU therapy, clinical presentation, histopathological findings, and treatment outcomes).
  - Articles available in English or with an English abstract.
  - Studies retrieved from PubMed, Scopus, and Google Scholar using relevant keywords (dermatomyositis, hydroxyurea, drug-induced dermatomyositis, hydroxyurea adverse effects, cutaneous toxicity, and hydroxyurea skin reactions).

Exclusion Criteria:
  - Studies focusing on classic autoimmune DM unrelated to drug exposure.
  - Cases of drug-induced DM associated with medications other than HU.
  - Reports lacking detailed individual case data (e.g., broader systematic reviews or meta-analyses without specific case details).
  - Non-human studies or experimental laboratory research.

After careful evaluation, we identified 27 relevant articles documenting similar cases of drug-induced DM associated with HU treatment.

The first reported case of HU-induced DM-like eruption dates back to 1989 when Marie Richard et al. [17] described a 55-year-old female with chronic myeloid leukemia (CML) and thrombocythemia who developed characteristic skin lesions on the face, dorsal hands, buccal mucosa, lips, and malleolus after four years of HU therapy. The eruption resolved following the discontinuation of HU and initiation of alternative chemotherapy [17]. After this initial report, the largest case series to first confirm this condition as a recognized adverse effect of HU treatment was published in 1995 by Sennett et al. [18], describing six patients with similar presentations. Among them, four had CML and two had essential thrombocythemia [18]. Skin lesions appeared after a median of 5.3 years of treatment, presenting as scaling erythema on the hands and feet, with no correlation to hematologic status. Muscle enzyme levels remained normal, and histopathology showed hyperkeratosis, vacuolar degeneration, and mild pericapillary inflammation. In three cases, lesions resolved within 1–6 months after stopping HU, while they persisted in patients who continued treatment. This study was the first to suggest a distinct drug-induced DM-like reaction linked to long-term HU use. However, since Sennett’s report, multiple similar cases have been reported and a 2024 review analyzing 134 studies identified HU as the most frequently implicated medication in drug-induced DM, accounting for 50 cases (30.3%) [19]. However, in our literature search, we identified only 21 published papers with 23 total patient cases, with sufficient clinical details to be included in our analysis. This discrepancy may be due to variations in reporting standards, keywords used for database queries, differences in study inclusion criteria, or unpublished cases referenced in broader reviews without full case descriptions. Additionally, studies that documented more than two patients were analyzed separately in a dedicated section. For the structured case summary, we analyzed only single- and two-patient case reports, as they provided detailed individual-level clinical data, allowing for a standardized comparison of demographic characteristics, latency periods, cutaneous involvement, histopathological findings, and treatment outcomes. Multi-patient studies, while valuable, often report aggregate data or ranges rather than specific case-by-case details, making them less suitable for inclusion in this quantitative analysis. However, these larger studies were reviewed separately to provide additional context on broader trends.

The median patient age was 67 years, with a slight predominance of females (13 out of 23 cases). The latency period from HU initiation to skin eruption ranged from 1 month to 15 years, with a median onset of 5 years [17,20,21,22,23,24,25,26,27,28,29,30,31,32,33,34,35,36,37,38,39].

The most commonly affected sites included the dorsal hands (21 cases, 91.3% of cases), periorbital region (10 cases, 43.5% of cases, heliotrope sign), and interphalangeal joints (16 cases, 69.6% of cases), though some cases also involved the face (12 cases), forearms (8 cases), retroauricular region (3 cases), trunk (5 cases), and lower extremities (7 cases). Out of the 23 patients, only 3 patients had just one affected site. Patients exhibited violaceous plaques, scaling erythema, periungual involvement, xerosis, poikiloderma, and plantar keratoderma. A subset of cases (6 out of 23) also developed non-melanoma skin cancers, and 5 cases reported chronic skin ulcers. Importantly, no patients had muscle involvement, and all had negative autoimmune markers, including ANA, CK, and aldolase [17,20,21,22,23,24,25,26,27,28,29,30,31,32,33,34,35,36,37,38,39].

HU discontinuation was the most effective treatment, leading to the gradual resolution of skin lesions in 21 out of 23 cases. Resolution times varied between 10 days and 10 months, though some lesions persisted despite dose reduction (three cases) or even after discontinuation. Alternative treatments, such as anagrelide (two cases) and busulfan (three cases), were used in some patients. Topical corticosteroids were commonly used as adjunctive therapy, but HU withdrawal remained the primary intervention [17,20,21,22,23,24,25,26,27,28,29,30,31,32,33,34,35,36,37,38,39]. For full details of reported cases, please refer to Appendix A, Table A1.

Across the reviewed studies, the histopathological features of HU-induced DM-like eruptions were largely consistent, with only minor variations in the degree of basal vacuolar degeneration, dermal mucin deposition, and perivascular lymphocytic infiltration. Given this uniformity, a comparative histopathological analysis between drug-induced and autoimmune dermatomyositis was not deemed necessary for this study. Additionally, due to the lack of significant differences, we did not include specific histopathological details for each case in our summary table, as they remained largely homogeneous across reports.

#### 3.2.1. Key Findings

In our review of 23 cases of HU-induced DM-like eruptions, the median latency period was 5 years (range: 1 month–15 years). HU discontinuation led to improvement in 91.3% of cases, with a median resolution time of 3 months. The results are summarized in Table 2.

#### 3.2.2. Summary of Treatment Outcomes

In terms of treatment outcomes, HU discontinuation was the most effective intervention, leading to the resolution of skin lesions in 91.3% of cases. Two cases (8.7%) underwent dose reduction without complete cessation, but lesions persisted. Topical corticosteroids were used adjunctively in all cases, though they did not prove sufficient as a standalone treatment. Systemic corticosteroids were employed in 52.2% of cases, primarily for moderate-to-severe presentations, and steroid-sparing agents (azathioprine and methotrexate) were introduced in 21.7% of cases to facilitate corticosteroid tapering. The median resolution time post-HU withdrawal was approximately 3 months, though ranges varied significantly based on treatment strategies. The results are presented in Table 3.

### 3.3. Case Series and Multi-Patient Studies

The selection process for this section followed the same methodology as described in the previous section. The same database queries and keyword searches were used to identify relevant multi-patient case series. Studies were included if they documented multiple cases of HU-induced DM-like eruptions with sufficient clinical details.

Based on the data presented in the table, we identified four case series describing HU-induced DM-like eruptions, with a total of 28 patients (Appendix A, Table A2). The mean patient age across all studies varied significantly, ranging from 43 years to 69 years, with individual age ranges spanning from 16 to 73 years. There was no strong sex predilection, with a fairly balanced distribution of males and females across the studies [8,18,40,41].

Regarding the underlying hematological conditions, CML was the most common diagnosis, accounting for 24 out of 28 cases (85.7%), while essential thrombocythemia was reported in 4 patients (14.3%). The mean latency period between HU initiation and the onset of DM-like skin eruptions varied significantly among studies [8,18,40,41]. Sennett et al. [18] and Yu et al. [40] reported a longer median onset period of approximately 5.3 and 5.5 years, respectively, while Oh et al. [41] and Vassallo et al. [8] documented much shorter median latencies of 1 and 2.5 years. Notably, the latency period varied widely across patients, ranging from as early as 6 months to as long as 10 years.

The cutaneous manifestations were diverse but consistent with classic DM-like features. Sennett et al. documented lesions primarily on the interphalangeal and metacarpophalangeal joints of the hands, as well as the dorsal feet. Yu et al. [40] reported hallmark DM skin findings, including heliotrope rash, poikiloderma, Gottron’s sign, and mechanic’s hands. In contrast, Oh et al. [41] described melanonychia in all patients, with two developing DM-like eruptions, but did not specify their exact locations. Vassallo et al. [8] reported more extensive cutaneous involvement, including acral erythema, xerosis, ichthyosiform changes, telangiectasia, and ulcers of both heels.

Case management and treatment outcomes varied among studies. Sennett et al. [18] reported that lesion resolution occurred in three patients within 1 to 6 months after HU discontinuation, whereas lesions persisted in two patients who continued therapy. Oh et al. [41] described mild improvement with topical and intralesional corticosteroids after one month but did not mention whether HU was discontinued. In contrast, Vassallo et al. [8] highlighted that HU discontinuation in all patients led to lesion resolution, with an average healing time of 9 months (range 4–24 months). Yu et al. [40] did not specify treatment details or outcomes.

While the latency period is variable, lesions predominantly affect acral and photo-exposed areas and frequently present with hallmark DM skin changes. Importantly, HU withdrawal appears to be the most effective intervention, leading to resolution in most cases, though the time to improvement remains variable [8,18,40,41].

### 3.4. Associations with Cutaneous Dysplasia and Skin Malignancies

Two studies [25,31] have reported cases of dermatomyositis-like eruptions (DM-LEs) associated with HU, in which histopathological analysis revealed dysplastic keratinocytes and p53 mutation expression within the lesions. Some of these patients subsequently developed multiple non-melanoma skin cancers (NMSCs) [25,31]. Similarly, HU-associated squamous dysplasia (HUSD) was first identified by Sanchez-Palacios et al. [35] in 2004 as a premalignant condition characterized by p53 mutation expression, serving as a potential precursor to multiple squamous cell carcinomas (SCCs). Since then, several additional cases of HUSD have been documented. Notably, all individuals diagnosed with HUSD exhibited skin rashes that bore some resemblance to DM-LEs but displayed more pronounced actinic damage and differed from classic DM-related rashes [35,42,43]. While Sanchez-Palacios [35] proposed that HUSD represents a distinct pathological entity separate from DM-LE, Kalajian et al. [31] suggested that both conditions may be part of a shared HU-induced phototoxic process. This ongoing debate raises the question of whether HUSD and DM-LE should be viewed as a continuum of the same disorder or as two independent conditions. Kalajian et al. [31] further argued that HUSD may be a more precise term, as it highlights the underlying neoplastic potential, whereas a DM-LE has historically been regarded as a benign phenomenon. Nevertheless, they recommended that HU-induced DM-LEs should be considered a possible premalignant stage leading to HU-related NMSCs, warranting careful monitoring and, in some cases, discontinuation of HU therapy.

Multiple studies have highlighted the potential premalignant nature of a DM-LE, challenging its previous classification as a benign condition. Consequently, in addition to discontinuing HU, close dermatologic monitoring is essential, with a proactive approach to biopsy any ulcerated lesions to exclude malignancy. Given the risk of neoplastic transformation, persistent ulcerative lesions should be carefully evaluated [31,44,45]. Given the premalignant potential of HU-related skin lesions, long-term dermatologic surveillance is essential for early detection and management of malignant transformation. Regular skin examinations, including dermoscopic evaluation and histopathologic assessment of suspicious lesions, should be conducted in patients with persistent or recurrent cutaneous manifestations. Moreover, proactive monitoring is particularly important in individuals with prolonged HU exposure, as cumulative drug effects may contribute to an increased risk of non-melanoma skin cancers.

If aggressive squamous dysplasia is detected, clinicians should be aware of the potential role of HU in its progression, and alternative treatments should be explored. While most patients show improvement following HU discontinuation, ongoing dermatologic surveillance remains essential [35]. Given that squamous dysplasia serves as a precursor to multiple invasive squamous cell carcinomas in sun-exposed areas, it should be recognized as part of the established spectrum of HU-related cutaneous toxicities [35].

The potential chemopreventive role of retinoids in managing squamous dysplasia warrants further investigation, particularly for patients requiring continued HU therapy. Low-dose oral retinoids have demonstrated efficacy in reducing cutaneous malignancies in immunosuppressed transplant recipients and may be a viable option for patients with chronic HU exposure and squamous dysplasia [46,47,48,49]. Emerging treatment modalities, such as retinoid therapy, have shown promise in reducing the risk of malignant transformation in patients with chronic HU-induced skin changes. Retinoids, including acitretin and isotretinoin, have been successfully used as chemopreventive agents in high-risk populations, such as organ transplant recipients and individuals with extensive actinic damage. Their potential role in reducing HU-related cutaneous dysplasia warrants further investigation, particularly in patients requiring prolonged HU therapy where discontinuation is not feasible [49].

## 4. Conclusions

An HU-induced DM-like eruption is a very rare but increasingly recognized cutaneous adverse effect of long-term HU therapy. Our case, along with the review of published reports, highlights key clinical features of this reaction, including its characteristic cutaneous presentation, absence of muscle involvement, and lack of myositis-specific antibodies. The latency period before eruption varies widely, emphasizing the need for continued dermatologic surveillance in patients undergoing prolonged HU therapy.

Management primarily involves the discontinuation of HU, which has been associated with lesion resolution in most cases. However, residual skin atrophy and chronic dermatologic complications, including non-melanoma skin cancers, may persist in some individuals. Alternative therapies such as anagrelide or busulfan may be necessary in cases where HU withdrawal is not feasible due to hematologic disease control.

Given the potential for underreporting and misdiagnosis, greater awareness among clinicians is essential to facilitate early recognition and appropriate management. Future research is needed to further elucidate the underlying pathophysiology and to optimize treatment strategies for affected patients. Moreover, future research should focus on mechanistic studies to elucidate why only a subset of patients on long-term HU therapy develop DM-like eruptions, potentially exploring genetic predispositions, immune system interactions, and individual variations in drug metabolism.

## Figures and Tables

**Table 1:** Comparison of HU-induced DM-like eruption vs. classic autoimmune DM.

Feature	Hydroxyurea-Induced DM-like Eruption	Classic Autoimmune Dermatomyositis
Etiology	Drug-induced (hydroxyurea exposure)	Autoimmune-mediated, often associated with myositis-specific antibodies
Cutaneous Manifestations	DM-like rash (Gottron’s papules, heliotrope rash, poikiloderma, periungual changes)	Similar rash distribution but often more widespread
Muscle Involvement	Absent	Present in most cases, with proximal muscle weakness
Serological Markers	Negative for myositis-specific antibodies	Often positive for anti-Mi-2, anti-TIF1-γ, anti-MDA5, anti-Jo-1 antibodies
Histopathology	Interface dermatitis, basal vacuolar degeneration, dermal mucin deposition	Similar findings, but often with more prominent inflammation and vasculopathy
Association with Malignancy	Possible, particularly with long-term HU exposure	Strong association, particularly with anti-TIF1-γ positive cases
Treatment	Discontinuation of HU, corticosteroids, immunosuppressants if needed	Systemic corticosteroids, immunosuppressants, IVIG, and other DM-specific therapies
Prognosis	Good with HU discontinuation; lesions usually resolve within months	Variable; can be chronic and relapsing, especially with muscle involvement

**Table 2:** Key findings from single- and two-patient case reports.

Feature	Findings
Total number of cases reviewed	23 cases
Mean patient age (years)	67 years
Female-to-male ratio	13:10
Median latency period (years)	5 years
Most commonly affected sites	Dorsal hands, face, periungual region
Percentage with periorbital involvement	43.5% (10/23 cases)
Percentage with dorsal hand involvement	91.3% (21/23 cases)
Percentage with nail involvement	69.6% (16/23 cases)
Percentage with histopathological DM-like features	100%
Percentage who discontinued HU	91.3% (21/23 cases)
Median resolution time after HU discontinuation (months)	3 months (range: 10 days–10 months)

**Table 3:** Summary of treatment outcomes.

Treatment Approach	Number of Cases	Outcome
HU discontinuation	21/23 cases (91.3%)	Complete or near-complete resolution
Dose reduction without discontinuation	2/23 cases (8.7%)	Lesions persisted but partially improved
Topical corticosteroids alone	All cases received topical corticosteroids	Used adjunctively; limited standalone efficacy
Systemic corticosteroids	12/23 cases (52.2%)	Accelerated improvement; used in moderate–severe cases
Steroid-sparing agents (e.g., azathioprine and methotrexate)	5/23 cases (21.7%)	Allowed steroid tapering; prevented relapses in some cases

## Data Availability

Not applicable.

## Abbreviations

HU	Hydroxyurea
DM	Dermatomyositis
MPDs	Myeloproliferative disorders
CBC	Complete Blood Count
CRP	C-Reactive Protein
ESR	Erythrocyte Sedimentation Rate
CK	Creatine Kinase
ANAs	Antinuclear Antibodies
EMG	Electromyography
H&E	Hematoxylin and Eosin
AB-PAS	Alcian Blue–Periodic Acid Schiff
DLQI	Dermatologic life quality index
AST	Aspartate aminotransferase
ALT	Alanine transaminase
LDH	Lactate dehydrogenase
MSAs	Myositis-specific autoantibodies
MAAs	Myositis-associated antibodies
MRI	Magnetic resonance imaging
PDM	Paraneoplastic dermatomyositis
CML	Chronic myeloid leukemia
DM-LE	Dermatomyositis-like eruption
NMSCs	Non-melanoma skin cancers
HUSD	Hydroxyurea-associated squamous dysplasia
SCCs	Squamous cell carcinomas

## Appendix A

**Table A1:** Summary of reported cases of hydroxyurea-induced dermatomyositis-like eruption.

Article	Patient Age/Sex	Underlying Condition	Time Since HU Initiation Until Skin Eruption	Site Affected	Case Management
Richard M et al. [17]	55/F	CML and thrombocythemia	4 years	Face, dorsal hands, buccal mucosa, lips, malleolus ulcer	HU discontinued, treated with alternative chemotherapy
Jenerowicz D et al. [20]	74/M	Polycythemia rubra vera	2 years	Hands (MCP and PIP joints), periorbital area	HU discontinued, clinical resolution in 3 months
Veraitch O et al. [21]	40/M	Polycythemia vera	1 month	Neck (C2-C3 dermatomes)	HU discontinued, CK improved in 2 months
Bahadoran P et al. [22]	62/M	CML	5 years	Face, hands, plantar keratoderma	HU discontinued, skin lesions faded within months
Bahadoran P et al. [22]	58/M	CML	7 years	Face, hands, elbows, legs	HU discontinued, moderate improvement
Dacey M et al. [23]	77/F	CML	5 years	Hands (Gottron’s papules, mechanic’s hands)	HU discontinued, resolution within 6 weeks
Daoud MS et al. [24]	69/M	CML	4 years	Dorsal hands, palms, elbows, dorsal feet	HU discontinued, improvement in 3 months
de Unamuno-Bustos B et al. [25]	76/F	Idiopathic myelofibrosis	4 years	Dorsal hands, face, retroauricular region, periungual area, malleolus	HU discontinued, replaced with anagrelide, resolution in 10 months
Elliott R et al. [26]	53/M	Polycythemia rubra vera	3 years	Dorsal hands	HU discontinued, skin started improving
Ito E et al. [27]	69/M	Essential thrombocytosis	5 years	Dorsal hands, foot ulcers	HU dose reduced, lesions persisted, foot ulcer improved slightly
Neill B et al. [28]	67/M	Polycythemia vera	15 years	Dorsal hands, head, trunk, extremities	HU discontinued, improvement noted
Haniffa MA et al. [29]	52/F	Polycythemia rubra vera	5 years	Leg ulcers, facial rash, interphalangeal joints	HU discontinued, ulcers and skin lesions resolved
De Unamuno B et al. [30]	76/F	Idiopathic myelofibrosis	4 years	Dorsal hands, face, neck	HU discontinued, replaced with anagrelide, resolution in 10 months
Kalajian AH et al. [31]	82/F	Dermatomyositis	4 years	Dorsal hands	HU discontinued, treated with immunosuppressants
Ahdoot R et al. [32]	84/F	JAK2 + myeloproliferative neoplasm	12 years	Dorsal hands	HU discontinued, skin biopsy confirmed dermatomyositis-like eruption
Moreno-Artero E et al. [33]	63/F	Essential thrombocythemia	3 years	Dorsal hands, feet, elbows, knees, presternal area	HU continued, lesions persisted
Nofal A et al. [34]	68/F	CML	6 years	Dorsal hands, face, nails, tongue, soles, heels	HU stopped, replaced by busulfan, improvement within 3–6 months
Sanchez-Palacios C et al. [35]	69/M	Essential thrombocythemia	9 years	Face, forearms, back of hands	HU discontinued, failed trial of anagrelide, restarted HU
Sanchez-Palacios C et al. [35]	66/M	CML	11 years	Scalp, multiple cutaneous cancers	HU continued, isotretinoin started
Slobodin G et al. [36]	57/F	CML	3 years	Dorsal hands, forearms	HU discontinued, patient died of coronary event
Suehiro M et al. [37]	52/F	CML	5 years	Dorsal hands, fingers, telangiectasia, ulcers	HU discontinued, improvement within 4 weeks
Varma S et al. [38]	81/F	Psoriasis	59 months	Dorsal hands, periungual telangiectasia	HU continued, lesions persisted
Zappala TM et al. [39]	66/F	Myelofibrosis	5 years	Dorsal hands, severe xerosis, palmar erythema, ulcers	HU discontinued, slow improvement over 6 months

**Table A2:** Summary of reported case series of hydroxyurea-induced dermatomyositis-like eruption.

Article	Number of Cases	Patient Age/Sex	Underlying Condition	Time Since HU Initiation Until Skin Eruption	Site Affected and Case Management
Vassallo C et al. [8]	7	Mean age 57 (range 25–73) 5 M/2 F	7/7 CML	2.5 years (range 7 to 60 months)	Acral erythema, xerosis, ichthyosiform lesions, telangiectases, ulcers of both heels, DM-like changes. HU was discontinued in all patients; resolution of skin lesions in 9 months average (range 4–24 months).
Sennet P et al. [18]	6	Mean age 69 (range 47–66) 3 M/3 F	4/6 CML 2/6 essential thrombocythemia	5.3 years (range 2–10)	Interphalangeal and metacarpophalangeal joints of the hands, and on the dorsal region of the feet. HU withdrawal led to lesion resolution within 1 to 6 months in three cases, while lesions persisted in two patients who continued treatment.
Yu K et al. [40]	8	Mean age 68 (range not specified) 4 M/4 F	CML and thrombocythemia	5.5 years (range not specified)	Heliotrope sign, poikiloderma, Gottron sign, mechanic’s hands. Not specified.
Oh ST et al. [41]	7	Mean age 43 (range 16–54) 4 M/3 F	7/7 CML	1 year (range 6 months to 4 years)	Melanonychia in all patients; two developed DM-like eruptions on sites not specified. Topical and intralesional corticosteroids, with mild improvement after 1 month.
